# Microcirculatory alterations induced by sedation in intensive care patients. Effects of midazolam alone and in association with sufentanil

**DOI:** 10.1186/cc5128

**Published:** 2006-12-15

**Authors:** Veronique Lamblin, Raphael Favory, Marie Boulo, Daniel Mathieu

**Affiliations:** 1 Service d'Urgence Respiratoire et Réanimation Médicale et de Médecine Hyperbare, Hôpital Calmette, Centre Hospitalier Universitaire, Boulevard du Professeur Jules Leclercq, 59037 Lille Cedex, France

## Abstract

**Introduction:**

Sedation is widely used in intensive care unit (ICU) patients to limit the risk of pulmonary barotrauma and to decrease oxygen needs. However, adverse effects of cc5128sedation have not been fully evaluated; in particular, effects of benzodiazepine and opiates on microcirculation have not been extensively studied. The aim of this study was to evaluate the microcirculatory effects of a sedation protocol commonly prescribed in the ICU.

**Methods:**

Ten non-septic patients under controlled ventilation requiring sedation for therapeutic purposes were enrolled in a prospective observational study conducted in an ICU of a university hospital. Sedation was conducted in two successive steps: first, each patient received midazolam (0.1 mg/kg per hour after a bolus of 0.05 mg/kg, then adapted to reach a Ramsay score of between 3 and 5). Second, after one hour, sufentanil was added (0.1 μg/kg per hour after a bolus of 0.1 μg/kg). Arterial pressure, heart rate, cardiac output determined by transthoracic impedance, transcutaneous oxygen (tcPO_2_) and carbon dioxide (tcPCO_2_) pressures, and microcirculatory blood flow determined by laser Doppler flowmetry at rest and during a reactive hyperaemia challenge were measured before sedation (NS period), one hour after midazolam infusion (H period), and one hour after midazolam-sufentanil infusion (HS period).

**Results:**

Arterial pressure decreased in both sedation periods, but heart rate, cardiac output, tcPO_2_, and tcPCO_2 _remained unchanged. In both sedation periods, microcirculatory changes occurred with an increase in cutaneous blood flow at rest (H period: 207 ± 25 perfusion units [PU] and HS period: 205 ± 25 PU versus NS period: 150 ± 22 PU, *p *< 0.05), decreased response to ischaemia (variation of blood flow to peak: H period: 97 ± 16 PU and HS period: 73 ± 9 PU versus NS period: 141 ± 14 PU, *p *< 0.05), and attenuation of vasomotion.

**Conclusion:**

Sedation with midazolam or a combination of midazolam and sufentanil induces a deterioration of vasomotion and microvascular response to ischaemia, raising the question of whether this effect may further alter tissue perfusion when already compromised, as in septic patients.

## Introduction

Because of its role in blood-tissue exchanges, the microcirculation is a fundamental element of the vascular network [1,2]. It has been long to recognise. It was only recently recognised that numerous pathologic conditions like arteriosclerosis, arterial hypertension, or diabetes alter the microcirculation, explaining, at least in part, the observed tissue hypoxia [3,4]. More recently, sepsis, a major cause of death in the intensive care unit (ICU), has been shown to induce microcirculatory dysfunction, even in its early stage and in the absence of circulatory failure [5]. These microcirculatory abnormalities compromise tissue oxygenation and may contribute to organ failure development [6-9].

Less attention has been paid to microcirculatory effects of treatments used in the ICU, in particular sedation. In the ICU, patients are often sedated in order to limit the risk of pulmonary barotrauma and to decrease oxygen needs. Sedation also facilitates daily care and diagnostic or therapeutic measures, often painful for these patients. It guarantees the safety of restless patients and improves their comfort. Due to the importance of this therapy, guidelines have been issued [10-12]. However, the consequences of drugs used for sedation have not been fully evaluated; in particular, the microcirculatory effects of benzodiazepine and opiates, the more commonly used drugs for ICU sedation, have not been extensively studied. The aim of our study was to evaluate the microcirculatory effects of a commonly prescribed sedation protocol, first by using a benzodiazepine alone (midazolam) and, second, by using a combination of midazolam and an opiate (sufentanil) in ICU non-septic patients.

## Materials and methods

### Patients

This study was prospectively conducted during a six month period in the ICU of the Calmette University Hospital (Lille, France) after approval by our local ethics committee. Informed written consent was obtained from each patient or the closest relative. The study population included ten non-septic patients under controlled ventilation for an acute respiratory failure and requiring sedation in order to optimise mechanical ventilation. In all patients, hypovolaemia had been either previously excluded or corrected by a fluid challenge. The haemodynamic status was stable for at least two hours before the beginning of the study. Patients treated with drugs known to alter microcirculation, such as inotropic, vasopressor, or vasodilator drugs, were excluded. Other exclusion criteria were sepsis, left ventricular dysfunction, cardiac arrhythmia, peripheral arterial disease, haemoglobin level of less than 8 g/dl, renal or hepatic failure, and all pathologic conditions known to be associated with microcirculation abnormalities.

Initially, patients were under controlled ventilation without any sedation for at least 24 hours. Patients were evaluated for study enrolment when the physician in charge decided to prescribe sedation. Once the patient was included, a complete set of measurements was obtained before sedation was prescribed (NS period). Then, sedation was conducted according to our routine protocol. First, patients received midazolam (0.1 mg/kg per hour after an intravenous bolus of 0.05 mg/kg) to reach a level of sedation ranging between 3 and 5 on the Ramsay scale. If sedation was considered insufficient after 20 minutes, a new bolus of 0.025 mg was injected and the injection rate was increased by 0.05 mg/kg per hour. In case of hypotension (systolic arterial pressure of less than 90 mm Hg), injection rate was decreased by 0.025 mg/kg per hour and colloids were infused until the hypotension was corrected. After one hour of sedation and when the targeted level of sedation was reached, a second set of measurements was obtained (H period). Second, sufentanil (0.1 μg/kg per hour after a bolus of 0.1 μg/kg) was added to midazolam (0.1 mg/kg per hour). If sedation was insufficient after 20 minutes, a new bolus of 0.05 μg was injected and the injection rate increased by 0.05 μg/kg per hour. In case of hypotension or bradycardia, the injection rate was decreased by 0.05 μg/kg per hour and colloid infusion (25 ml/minute) was started. The targeted level of sedation and recordings were the same as for the preceding step (HS period).

### Measurements

A complete set of measurements included heart rate (HR), mean arterial pressure (MAP), percutaneous oxygen saturation (SpO_2_), cardiac output (CO), transcutaneous oxygen (tcPO_2_) and carbon dioxide (tcPCO_2_) pressures, and cutaneous blood flow at rest (Φrest) and during hyperaemia. CO was measured by transthoracic impedance (Bomed NCCOM 3; Bomed, Irvine, CA, USA) using a lateral spot electrode configuration and incorporating the Sramek-Bernstein equation [13]. The mean of five consecutive determinations of CO was recorded as CO. A satisfactory agreement between this non-invasive method and thermodilution had been observed in critically ill patients under mechanical ventilation, and reproducibility is comparable to reference techniques [14,15]. tcPO_2 _and tcPCO_2 _were continuously recorded (Kontron Instruments, Basel, Switzerland).

Cutaneous blood flow was measured with a laser Doppler flowmeter probe and device (Periflux PF4; Perimed AB, Stockholm, Sweden). This technique allows real-time and continuous monitoring suitable for cutaneous microcirculation inquiries [16,17]. Laser Doppler flowmetry (LDF) had been previously validated in animals and humans by the thermal clearance technique, *in vivo *microscopy, and plethysmography [18,19]. Different signals are available: velocity, concentration of moving blood cells (CMBC), and their product, flow. Cutaneous blood flow was measured at rest and during reactive hyperaemia, and values were expressed in perfusion units. The laser Doppler signal was continuously registered on a personal computer. The gain was adjusted to 1, the cutoff frequency to 12 Hz, and the time constant to 0.2 seconds. A constant back-scattered light of at least 30% of the emitted light indicates an adequate contact of the optical probe with the tissue surface. Before each patient was studied, a calibration based on the random Brownian motion of small scatterers in an emulsion (Periflux motility standard; Perimed AB) was performed. Φrest, velocity, and CMBC were taken as the means of a five minute stable LDF recording [20].

**Figure 1:**
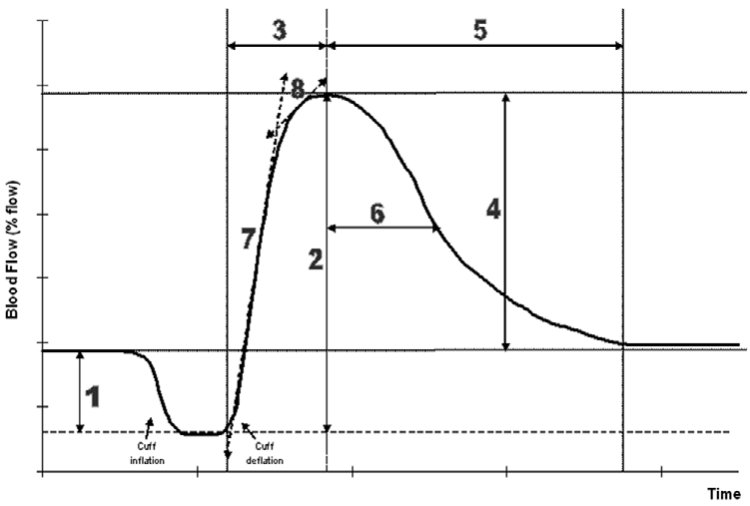
Schematic representation of reactive hyperaemia and measurements realised from laser Doppler recording. 1: Mean blood flow at rest (Φrest). 2: Peak flow (Φpeak). 3: Time to peak. 4: ΔΦ = Φpeak - Φrest. 5: Time to flow normalisation. 6: Time to half flow normalisation. 7: First upward slope calculated for the first 3 seconds. 8: Second upward slope calculated for the second half.

**Figure 2:**
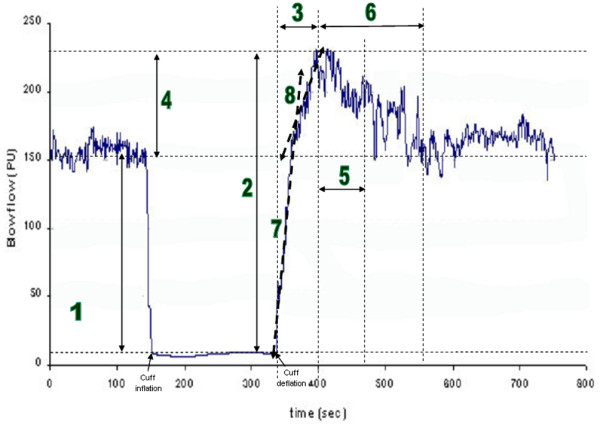
Example of a laser Doppler recording of blood flow during reactive hyperaemia in a patient sedated with midazolam. 1: Mean blood flow at rest (Φrest). 2: Peak flow (Φpeak). 3: Time to peak. 4: ΔΦ = Φpeak - Φrest. 5: Time to flow normalisation. 6: Time to half flow normalisation. 7: First upward slope calculated for the first three seconds. 8: Second upward slope calculated for the second half. PU, perfusion units.

Reactive hyperaemia was produced by an arrest of forearm blood flow with a pneumatic cuff inflated to a suprasystolic pressure of 200 mm Hg for three minutes. The signal obtained during this complete arterial occlusion is flux-independent and is taken as the biological zero for blood flow measurements before and during the subsequent reactive hyperaemia manoeuvre. On completion of the ischaemic period, the occluding cuff was rapidly deflated to zero. Peak flow was defined as the highest flow signal during the postocclusive phase. Reactive hyperaemia is considered to test organ maximal ability to increase flow on demand, when demand has been maximally stimulated by a zero flow. This manoeuvre is widely used as a vascular reactivity test [21,22]. The following were measured on LDF recordings: maximum flow during the reactive hyperaemia peak (Φpeak), flow variation (ΔΦ = Φpeak - Φrest), time to peak (Tpeak), time to half flow normalisation (T_1/2 _R), and time to flow normalisation. On each curve of reactive hyperaemia, the slope of the best-fit line traced using linear regression associated with the upward portion of hyperaemia peak (first three seconds: slope 1; second half: slope 2) was determined. All of these parameters have been shown to be reproducible [23] and are represented in Figure 1. An example of an LDF recording is shown in Figure 2.

During the whole study period, patients remained in a constant supine position in comfortable environmental conditions that were maintained without change. The tcPO_2 _and tcPCO_2 _probe was placed on the forearm skin distal to the cuff. The LDF probe remained placed on the same location (left mean finger pad) without any displacement.

### Vasomotion

The small arteries of the microcirculation present rhythmic and spontaneous variations of their diameter called, by convention, vasomotion and characterised by a frequency and amplitude [24]. This low-frequency rhythm is present in the cutaneous microcirculation and can be studied with LDF. According to the classification described by Colantuoni and colleagues [25], frequencies of vasomotion ranging between 0 and 3.5 cycles per minute (cpm) correspond to the A1 medium arteries, those ranging between 2.5 and 4.7 cpm to the A2 small arteries, those ranging between 4 and 7.6 cpm to the A3 small arteries, and those ranging between 7.6 and 12 cpm to the final arteries, A4.

Analysis by Fast Fourier Transformation allows the determination of the spectra of frequencies and amplitudes contained in the LDF signal for the frequencies ranging between 0 and 12 cpm at rest and during reactive hyperaemia (PSW Software; Perimed AB). At each frequency, an amplitude, defined as the importance of the studied frequency in the portion of LDF recording analysed, is associated. In each period and for each frequency (1 to 12 cpm), the median of the vasomotion amplitudes was determined.

### Statistical analysis

In our study, each patient acted as his or her own control. All data are expressed as means ± standard error of the mean except for the frequencies of vasomotion expressed as a median. Repeated measures analysis of variance was used to compare NS, H, and HS periods. When significant, inter-group comparisons were made by Tukey's multiple comparison tests. Vasomotion frequency spectra were compared by a Kolmogorov-Smirnov test, according to sedation type, and then a non-parametric repeated measures analysis of variance (Friedman test) was used to compare NS, H, and HS periods for each vasomotion frequency studied (0 to 12 cpm). Significance was accepted at *p *< 0.05.

## Results

Ten patients were included in our study. General characteristics are summarised in Table 1. When the H-period data were collected, 26 ± 13 mg of midazolam had been infused and the Ramsay score obtained was 4 ± 1. When the HS-period data were collected, 41 ± 20 mg of midazolam had been infused during the two hour infusion and 55 ± 36 μg of sufentanil was added the second hour. The Ramsay score obtained was 5 ± 1.

### Pattern of resting parameters

MAP decreased significantly during the sedation periods (H and HS) compared to the NS period with no difference between H and HS periods. HR, CO, SpO_2_, tcPO_2_, and tcPCO_2 _remained unchanged in all periods. Mean blood flow at rest (Φrest) increased during the two sedation periods compared to the NS period. CMBC remained unchanged by sedation, whereas red blood cell velocity increased during H and HS periods compared to the NS period (Table 2).

**Figure 3:**
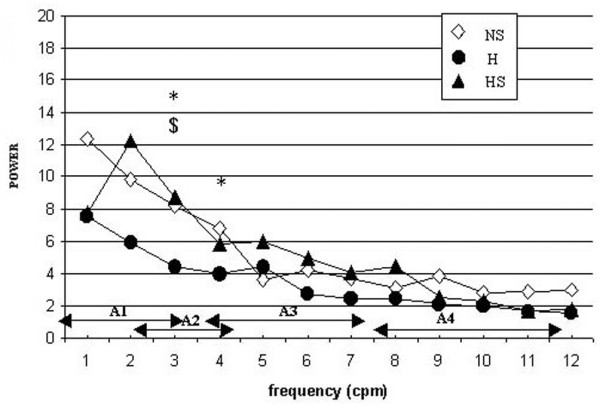
Distribution of vasomotion frequencies at rest. Kolmogorov-Smirnov test: *p *< 0.05 NS and HS periods versus H period. Friedman test: *p < 0.05 NS period versus H period, ^$^*p *< 0.05 HS period versus H period. cpm, cycles per minute; H period, set of measurements obtained when the patients were sedated by midazolam; HS period, set of measurements obtained when the patients were sedated by midazolam and sufentanil; NS period, set of measurements obtained when the patients were non-sedated.

Vasomotion frequency spectra obtained in each sedation period are represented in Figure 3. Distribution of vasomotion frequencies was significantly different during the H period compared to NS and HS periods. There was no difference between the NS and HS periods. Midazolam significantly decreased vasomotion, and sufentanil restored the vasomotion. Midazolam acted especially on the low-frequency vasomotion, which corresponds to the A1 and A2 small arteries (Figure 3).

### Reactive hyperaemia

Peak blood flow (Φpeak) remained unchanged during sedation periods versus the NS period. ΔΦ decreased significantly during H and HS periods versus the NS period, whereas no significant difference existed between sedation periods. Slope 1 associated with the initial upward portion of hyperaemia peak was not changed by midazolam but increased when sufentanil was added to midazolam. Slope 2 associated with the second upward portion of peak was not influenced by sedation (Table 3).

**Figure 4:**
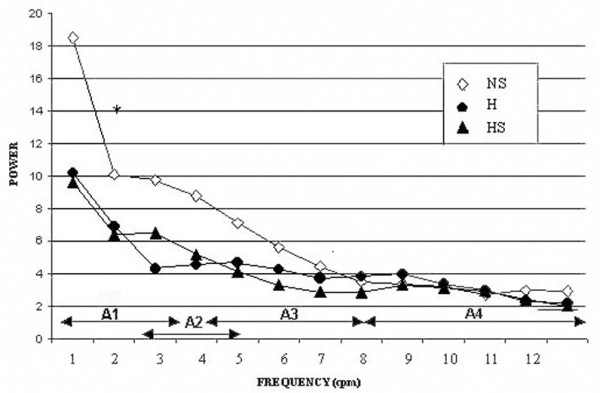
Distribution of vasomotion frequencies during reactive hyperaemia according to sedation. Kolmogorov-Smirnov test: *p < 0.05 NS period versus H and HS periods. cpm, cycles per minute; H period, set of measurements obtained when the patients were sedated by midazolam; HS period, set of measurements obtained when the patients were sedated by midazolam and sufentanil; NS period, set of measurements obtained when the patients were non-sedated.

In the NS period, vasomotion wave amplitudes were higher during reactive hyperaemia than at rest. This reinforcement of vasomotion by reactive hyperaemia has been described in the literature and proves that the microcirculation of our patients reacted normally [22]. In vasomotion frequency analysis, this phenomenon was observed mainly in the low frequencies (1 to 3 cpm) and thus concerned mainly the A1 small arteries. In contrast, during sedation periods, this inductive role of reactive hyperaemia was not observed. Vasomotion was depressed and this effect predominated in A1 small arteries (Figures 3 and 4).

## Discussion

Sedation is widely used in ICU patients but its potentially deleterious effects, in particular on the microvascular bed, have not been precisely evaluated. In this study, we found that sedation using midazolam or a combination of midazolam and sufentanil induces microcirculatory changes with increased cutaneous blood flow, decreased response to ischaemia, and attenuation of vasomotion.

### Effects of sedation on cutaneous microcirculation at rest

#### Microcirculation and midazolam

Sedation with midazolam induces a significant decrease of MAP. Cardiovascular effects of benzodiazepines are well known in anaesthesia [26,27]. However, with the subanaesthesic dose of benzodiazepine recommended for ICU sedation, MAP and HR decrease only slightly [28], as we have noted in our study.

Mean cutaneous blood flow increased after one hour of sedation by midazolam. In parallel to blood flow, the red blood cell velocity increased, whereas CMBC remained stable. These data are in favour of a cutaneous vasodilation induced by midazolam and are in agreement with the literature [29]. Studies of cutaneous and subcutaneous blood flows after injection of benzodiazepine show an increase in the surface cutaneous thermal clearance as well as a stability of the deep thermal clearance, corresponding to an increase in cutaneous blood flow with no deterioration of subcutaneous blood flow [30,31]. In another study, LDF also reveals an increase in cutaneous blood flow among anaesthetised and hypothermic patients compared to control subjects [32].

The increase in cutaneous blood flow may be explained by the direct vasodilator effect of benzodiazepines [29]. Midazolam attenuates the smooth muscle contraction induced by norepinephrine, acting by an inhibition of Ca^2+ ^influx occurring through voltage-operated Ca^2+ ^channels and through agonist-mediated Ca^2+ ^channels and by an inhibition of Ca^2+ ^release from intracellular storage sites (sarcoplasmic reticulum) [33]. Endothelium-dependent mechanisms also take part in the vasodilation produced by midazolam through the release of nitric oxide (NO) from vascular endothelium [34].

#### Microcirculation and the combination of midazolam and sufentanil

In our study, the combination of midazolam and sufentanil worsened hypotension (only slightly) and bradycardia but did not change CO. Φrest was higher during the HS period than during the NS period but was not different from that observed during the H period. Contradictory results concerning the effects of sufentanil on vascular tone have been described in the literature. Sufentanil has been shown to decrease peripheral vascular resistances through a direct vascular effect [35]. Karasawa and colleagues [36] showed this effect to be due to an endothelium-independent vasorelaxation mediated by both an alpha-receptor blockage and a direct effect on smooth muscle. In addition, Stefano and colleagues [37] reported that endothelial cells contain opiate receptors called mu3 which are coupled to NO release and vasodilation. On the other hand, a direct contractile effect on vascular smooth muscle has also been described [38]. As shown by Brookes and colleagues [39], the discrepancy between these two studies may be explained by differences in doses. In our study, sufentanil dose may have been insufficient to induce additional microcirculatory disturbances.

### Effects of sedation on cutaneous microcirculation response to ischaemia

#### Reactive hyperaemia

Reactive hyperaemia is a well-established and widely used challenge to test microcirculation reactivity. This method has been largely validated and is reproducible in humans [40,41]. It corresponds to an increase in local blood flow, secondary to a transient ischaemia, and is thought to exactly reflect the circulatory deficit that has occurred during the vascular occlusion.

Reactive hyperaemia is the result of the combination of several phenomena divided into a myogenic phase followed by a metabolic phase. The myogenic phase corresponds to the changes of arteriolar diameter in response to pressure modifications and is thought to be reflected by the initial upward portion (slope 1) of the hyperaemia peak [42]. At the time of the metabolic phase thought to be reflected by the second part of the upward portion (slope 2), the arteriolar vasodilation is the result of factors acting directly on the vascular smooth muscle or via the endothelium [42,43]. Engelke and colleagues [44] showed that prostaglandins, released from the vascular endothelium, are important determinants of the hyperaemia peak, in contrast to NO, which takes part only in the maintenance of the vasodilation after the peak [45].

#### Reactive hyperaemia and midazolam

In our study, midazolam did not influence the blood flow at hyperaemia peak. On the other hand, ΔΦ (Φpeak - Φrest) was decreased by 30% compared to the NS period. Peak blood flow represents the maximum microcirculatory blood flow obtainable by vasodilation. This explains the stability of Φpeak and the decrease of ΔΦ during reactive hyperaemia in patients during the H period, in whom an increased Φrest existed before the reactive hyperaemia manoeuvre. Time to peak tended to increase. All of these results show that midazolam induced a limitation of the vascular response to ischaemia.

#### Reactive hyperaemia and the combination of midazolam and sufentanil

During reactive hyperaemia, addition of sufentanil to midazolam did not change peak blood flow compared to NS and H periods. On the other hand, ΔΦ decreased by 50% during the HS period compared to the NS period but did not differ from the H period. Time to peak decreased during the HS period compared to the H period without reaching the threshold of significance.

The slope 1 was significantly increased compared to the H period, evoking modification of the myogenic phase of reactive hyperaemia. During the HS period, small arteries seemed to vasodilate more easily and more quickly than during the H period. Sufentanil could induce a decrease in the smooth vascular tonicity by acting directly on the vascular smooth muscle and making vasorelaxation easier. These results are in agreement with those of Karasawa and colleagues [36], who found that fentanyl induces vasodilation via a direct action on muscular smooth cell and by locking alpha-adrenergic receptors. However, because in our study these changes were observed during the injection of a combination of midazolam and sufentanil, we cannot determine whether sufentanil was, by itself, responsible for the decrease in vascular tonicity or only reinforced an effect started under midazolam.

### Effects of sedation on vasomotion

Blood flow in the microcirculation is not continuous but is subject to cyclic variations in which periods of high blood flow alternate with periods of no flow. This phenomenon has been called vasomotion and is due to changes in lumen diameters which result from periodic activity of muscle cells in the microvessel wall governed by oscillation of intracellular calcium concentration [46].

Vasomotion has been observed since the inception of microvascular studies by intravital microscopy [47,48]. Later, when LDF appeared, the oscillatory flow patterns observed were related to the vasomotion activity of the microcirculation. Subsequently, it was shown that frequency analysis of LDF recordings was able to discriminate between the types of vessels from which the signal originates and that low-frequency flow oscillations were directly related to vasomotion of the arterioles [25,49].

#### Vasomotion and sedation

In our study, we observed a significant reduction in the importance of cutaneous vasomotion at rest and during reactive hyperaemia in the group sedated with midazolam. The combination of midazolam and sufentanil seemed to restore cutaneous vasomotion to its resting level. Anaesthetic drugs have long been recognised to alter vasomotion [50,51].

Decrease of vasomotion observed during midazolam infusion is probably due to the benzodiazepine effects on intracellular calcium concentration: inhibition of Ca^2+ ^influx and decrease of Ca^2+ ^release from sarcoplasmic reticulum [33]. An explanation for the restoration of vasomotion when sufentanil is added to midazolam is less evident. Stephano and colleagues [37] have shown that opiates induce NO release through endothelial mu3 receptors. We hypothesise that this increase in NO could elevate cyclic guanosine monophosphate (cGMP) concentration in smooth muscle cells, thereby increasing the cGMP-dependent Ca^2+^-activated chloride channel, which has been shown to be responsible for coupling the Ca^2+ ^oscillations generated by the sarcoplasmic reticulum to the membrane current that synchronises individual cells [52,53].

In the NS period, we found vasomotion frequency distributions to be more important during reactive hyperaemia than at rest, evidence of a potentiation of vasomotion by hyperaemia. During midazolam infusion, an inhibition of this increase of vasomotion induced by reactive hyperaemia was noted. Frequency analysis of the LDF recordings showed that the action of midazolam on vasomotion prevailed on the A1 small arteries (frequency of between 1 and 3 cpm). On the contrary, Colantuoni and colleagues [25] found that the inhibition of vasomotion by anaesthesia concerns vessels of all orders. The discrepancy with our study may be explained by technical reasons. In our study, 70% of the LDF signal came from the largest arterioles, A1 and A2, and only 30% of the signal from the smallest arteries, A3 and A4 (Figures 3 and 4). Consequently, it may have been statistically easier to highlight an effect of sedation on the A1 small arteries even if sedation deteriorates the vasomotion in all four orders of small arteries.

Our study suffers from some limitations. First, the small number of patients may have hidden some true variations. Second, the study design did not include a randomisation between the two steps. So, a carry-over effect may interfere when studying the combination of midazolam and sufentanil. In accordance with the aim of our study, we designed our sedation protocol following widely accepted guidelines in order to be closer to routine clinical practice. Doses of sufentanil used were perhaps not sufficient to induce an additional effect on cutaneous microcirculation. In clinical practice, the amounts of opiates used are often higher than those recommended. So, sufentanil's own effects may have been minimised.

Third, we chose the LDF technique because it is non-invasive and easy to use in an ICU setting. Numerous techniques have been proposed to explore the microcirculation, none of which is without critics. Recently, a new technique, orthogonal polarisation spectral imaging, has been used in the ICU. It has several advantages, in particular in separating respective changes in small arteries, capillaries, and venules [8]. However, it gives semi-quantitative measurements, suffers from an intra/inter-observer variability of 5% to 10%, and is less suitable for monitoring short-term microcirculatory blood flow change as during recruitment manoeuvres. LDF is more suitable for monitoring such rapid microcirculatory blood flow changes but raises problems of calibration, artifact related to patient movements, inability to separate respective changes between all the vessels included in the investigated volume, and inter-individual flow variations [54]. In our series, we noted great inter-individual variations of blood flows at rest and during reactive hyperaemia. However, for the same patient, the signal is reproducible provided that the position of probe and conditions of measurement remain identical (haemodynamic, temperature) [23,55]. Cutaneous blood flow varies according to the area measured. Indeed, in the upper limb, the palms of the hand and finger pads are better vascularised than the forearm or the dorsum of the hand. We chose the pad of the mean finger as the site of recording because this zone is highly vascularised and, consequently, flow is more easily detectable by LDF [56]. Lastly, we studied the effects of sedation on cutaneous microcirculation. Even if skin preparations have often been used as a model to study microcirculation, extension of our results to other microcirculations may be made only with caution. Further studies have to be carried out to determine whether microcirculation in other organs reacts in the same way.

## Conclusion

Our study is one of the first to examine the effects of a sedation regimen commonly used in the ICU on cutaneous microcirculation. Benzodiazepine induces an increase in cutaneous blood flow secondary to vasodilation, a decrease in reactive hyperaemia, and alterations of vasomotion. Addition of sufentanil does not substantially modify the results obtained.

Clinical studies have clearly established that alterations of normal microcirculatory control mechanisms may compromise the tissue nutrient blood flow and may contribute to the development of organ failure in septic patients [9,57,58]. Our study raises the question of whether sedation with benzodiazepine or a combination of benzodiazepine and sufentanil by deteriorating vasomotion and vascular reactivity to ischaemia may further alter tissue perfusion when already compromised, as in septic patients.

## Key messages

• Sedation with midazolam alone or in association with sufentanil induces a deterioration of vasomotion and microvascular response to ischaemia. This raises the question of whether sedation may further alter tissue perfusion when already compromised, as in septic patients.

## Abbreviations

ΔΦ = flow variation during reactive hyperaemia (ΔΦ = Φpeak - Φrest); Φpeak = maximum cutaneous blood flow during the reactive hyperaemia peak; Φrest = cutaneous blood flow at rest; cGMP = cyclic guanosine monophosphate; CMBC = concentration of moving blood cells; CO = cardiac output; cpm = cycles per minute; H period = set of measurements obtained when the patients were sedated by midazolam; HR = heart rate; HS period = set of measurements obtained when the patients were sedated by midazolam and sufentanil; ICU = intensive care unit; LDF = laser Doppler flowmetry; MAP = mean arterial pressure; NO = nitric oxide; NS period = set of measurements obtained when the patients were non-sedated; SpO_2 _= percutaneous oxygen saturation; T_1/2 _R = time to half flow normalisation; tcPCO_2 _= transcutaneous carbon dioxide pressure; tcPO_2 _= transcutaneous oxygen pressure; Tpeak = time to reactive hyperaemia peak.

## Competing interests

The authors declare that they have no competing interests.

## Authors' contributions

VL conceived the protocol, participated in its design, carried out bedside measurements and documentation, and drafted the manuscript. MB and RF conceived the protocol and helped to interpret the data. DM conceived the protocol, participated in its design and coordination, and helped to interpret the data and to draft the manuscript. All authors read and approved the final manuscript.

## Figures and Tables

**Table 1:** General characteristics of study population

Patient	Age (years)	Gender	Weight (kg)	Temperature (°C)	Respiratory failure	SAPS II	Outcome
1	74	Male	70	36.9	COPD	34	Alive
2	67	Female	60	37.2	COPD	24	Alive
3	66	Male	84	37.1	Postoperative	35	Alive
4	77	Male	70	36.8	Postoperative	37	Dead
5	19	Female	80	36.8	Asthma	44	Alive
6	50	Female	70	37.4	COPD, obesity	36	Alive
7	76	Female	87	36.8	SAS, obesity	86	Alive
8	73	Male	73	36.9	COPD	38	Alive
9	57	Male	70	37.5	COPD	39	Alive
10	75	Male	80	37.0	COPD	47	Dead

COPD, chronic obstructive pulmonary disease; SAPS II, Simplified Acute Physiology Score II; SAS, sleep apnoea syndrome.

**Table 2:** Resting parameters

	NS period	H period	HS period
MAP (mm Hg)	94 ± 3	84 ± 4^a^	81 ± 4^a^
HR (beats per minute)	83 ± 6	81 ± 5	78 ± 5
CO (litres/minute)	5.38 ± 0.82	5.27 ± 0.82	5.33 ± 1.08
SpO_2 _(percentage)	96.4 ± 0.5	96.7 ± 0.5	96.1 ± 0.5
tcPO_2 _(mm Hg)	67 ± 6	61 ± 6	61 ± 7
tcPCO_2 _(mm Hg)	45 ± 2	44 ± 4	42 ± 4
Φrest (PU)	150 ± 22	207 ± 25^a^	205 ± 25^a^
CMBC (CU)	145 ± 15	152 ± 16	155 ± 15
Velocity (VU)	1.06 ± 0.11	1.39 ± 0.15^a^	1.37 ± 0.79^a^

^a^*p *< 0.05 versus NS period. Φrest, mean blood flow at rest; CMBC, concentration of moving blood cells in concentration units (CU); CO, cardiac output; H period, set of measurements obtained when the patients were sedated by midazolam; HR, heart rate; HS period, set of measurements obtained when the patients were sedated by midazolam and sufentanil; MAP, mean arterial pressure; NS period, set of measurements obtained when the patients were non-sedated; PU, perfusion units; SpO_2_, percutaneous oxygen saturation; tcPCO_2_, transcutaneous carbon dioxide pressure; tcPO_2_, transcutaneous oxygen pressure; velocity expressed in velocity units (VU), Φrest (perfusion units)/CMBC.

**Table 3:** Changes in Doppler measurements during reactive hyperaemia according to sedation types

	NS period	H period	HS period
Φpeak (PU)	292 ± 31	304 ± 28	274 ± 25
ΔΦ (PU)	141 ± 14	97 ± 16^a^	73 ± 9^a^
TP (seconds)	23.5 ± 4.3	43.8 ± 11.5	24.2 ± 8.2
T_1/2 _R (seconds)	38.8 ± 13.7	22.9 ± 3.4	23.8 ± 5.5
TR (seconds)	170.1 ± 40.5	117.2 ± 3.4	85.1 ± 14.9
Slope 1 (PU/second)	53.6 ± 13.3	51.5 ± 8.8	74.1 ± 10.9^a,b^
Slope 2 (PU/second)	10.8 ± 3.7	8.2 ± 3.0	8.7 ± 0.7

^a^*p *< 0.05 versus NS period; ^b^*p *< 0.05 versus H period. ΔΦ, Φpeak - Φrest; Φpeak, maximal blood flow during reactive hyperaemia; H period, set of measurements obtained when the patients were sedated by midazolam; HS period, set of measurements obtained when the patients were sedated by midazolam and sufentanil; NS period, set of measurements obtained when the patients were non-sedated; PU, perfusion units; T_1/2 _R, time to half flow normalisation; TP, time to peak; TR, time to flow normalisation.
